# Influence of involvement of anterior leaflet versus posterior leaflet on residual regurgitation as assessed by transesophageal echocardiography in patients undergoing valve repair for mitral regurgitation due to mitral valve prolapse

**DOI:** 10.1186/1476-7120-7-54

**Published:** 2009-11-17

**Authors:** Laureta Sulcaj, Antonio Rizza, Mattia Glauber, Giuseppe Trianni, Cataldo Palmieri, Marcello Ravani, Alban Dibra, Stefano Maffei, Sergio Berti

**Affiliations:** 1 G. Monasterio Foundation, CNR-Regione Toscana, Institute of Clinical Physiology, G. Pasquinucci Hospital, Massa, Italy; 2 University Hospital Center "Nene Tereza", Tirana, (LS, AD), Albania

## Abstract

**Background:**

Repair of anterior leaflet prolapse is technically more challenging and this might influence outcomes as compared to the repair of posterior leaflet prolapse in patients undergoing surgical correction of mitral regurgitation. We investigated the association of anterior leaflet prolapse with minor residual mitral regurgitation (MR) in patients with mitral valve prolapse (MVP) who underwent valve repair.

**Methods:**

Eligible for this study were consecutive patients with severe MR due to MVP, who underwent mitral valve repair with residual MR by postpump transesophageal echocardiography ≤2+ during a 20-month period at Pasquinucci Hospital, Massa. Patients undergoing other cardiovascular surgical interventions were excluded. Two groups were defined according to the involvement of mitral valve leaflets: group 1, consisting of patients with anterior leaflet prolapse (isolated or not); and group 2, consisting of patients with isolated posterior leaflet prolapse.

**Results:**

A total of 70 patients (18 in group 1 and 52 in group 2) were analyzed. Patients in group 2 were younger than those in group 1, but the difference was not significant (P = 0.052). There were no significant differences between the 2 study groups with respect to other variables. The proportion of patients with residual MR 1+/2+ was higher in group 1 than in group 2 (61.1% vs. 32.7%, respectively; P = 0.034). In a logistic regression model, anterior leaflet prolapse was an independent predictor of residual MR 1+/2+ (odds ratio, 4.0; 95% confidence interval, 1.14 to 14.04; P = 0.03).

**Conclusion:**

In our study population, patients with anterior leaflet prolapse had a higher proportion of residual MR 1+/2+ as compared to those with posterior leaflet prolapse after repair of mitral valve.

## Background

Mitral regurgitation is the most frequently valvular pathology encountered nowadays in clinical practice [1]. During the recent decades, there has been a major change not only in the etiology of mitral regurgitation which is now predominantly of degenerative and ischemic origin [2], but also in the non-invasive assessment of the severity and mechanisms responsible for mitral regurgitation by means of transesophageal echocardiography [1,3].

Surgical repair is the most widely used treatment strategy for the correction of mitral regurgitation caused by mitral valve prolapse [4]. Although repair of mitral valve is feasible in up to 95% of the patients with mitral regurgitation of degenerative origin [5], it has been traditionally accepted that prolapse of both mitral valve leaflets is associated with a more complicated repair procedure. On the other hand, repair of the prolapse of the anterior leaflet of mitral valve is technically more demanding and this might lead to less favourable results as compared to the repair of the prolapse of posterior leaflet [6].

Application of new repair techniques for the surgical correction of mitral regurgitation due to the prolapse of anterior leaflet has resulted effective and has positively influenced the postoperative outcome, despite a lack of standardization of the surgical approach. Evidence on the postoperative outcome after surgical repair of mitral regurgitation caused by involvement of anterior mitral leaflet as compared to the posterior mitral leaflet has been limited.

## Methods

### Objective

We sought to compare residual mitral regurgitation after repair of anterior leaflet (isolated or not) with residual mitral regurgitation after repair of posterior leaflet in patients undergoing valve repair due to mitral regurgitation caused by mitral valve prolapse.

### Source of information

Relevant information for this study was obtained from the database of patients admitted and treated at Ospedale Pasquinucci, IFC-CNR, Massa, Italy.

### Study population

Eligible for this study were all patients with severe mitral regurgitation due to mitral valve prolapse who underwent for the first time surgical repair of mitral valve and presented with residual mitral regurgitation ≤2+ as assessed by means of post pump transesophageal echocardiography from January 2004 to October 2005, at Ospedale Pasquinucci, Massa, Italy.

Excluded from this study were patients in whom the myocardial ischemia could have contributed mitral regurgitation as well patients who concomitantly underwent other cardiac or vascular surgical procedures such as aorto-coronary bypass surgery, other cardiac valve surgery, surgical interventions for disease of abdominal or thoracic aorta or patients who were surgically treated for cardiac congenital diseases. We did not impose any restriction criteria regarding clinical presentation, age, or accompanying medical conditions.

Two groups were defined according to the involvement of mitral valve leaflets: group 1, consisting of patients with anterior leaflet prolapse (isolated or not); and group 2, consisting of patients with isolated posterior leaflet prolapse.

This was a retrospective study. However, all data have been collected and entered into the hospital database in a prospective manner.

### Methodology used for the measurement of mitral regurgitation

We employed a qualitative assessment of the severity of mitral regurgitation [7]. Transthoracic echocardiography was performed with a Vivid 7 echo machine, a 3-MHz probe. Tranesophageal echocardiography was performed with Philips Sonos 7500 echo machine, a 3-MHz probe.

### Follow-up

All patients were examined by means of transthoracic and transesophageal echocardiography before and after intervention as well as before and after cardiopulmonary bypass. Physicians were unaware of the surgical result. Residual mitral regurgitation after cardiopulmonary bypass was graded from 1+ to 4+.

### Statistical analysis

All data presented are shown as mean ± standard deviation or numbers (percentages). To compare the differences between groups, we used student *t *test for continuous data and chi-square or Fisher's exact test for categorical data, as appropriate. To identify the independent role of anterior leaflet involvement on the residual mitral regurgitation after valve repair, a logistic regression model was build. Statistical significance was accepted for a two-tailed P < 0.05.

## Results

### Baseline clinical characteristics

A total of 70 patients (18 patients included in group 1 and 52 patients included in group 2) were analyzed. Thirty percent of the patients were women. Mean age of study patients was 63.5 ± 11.5 years old. Patients in group 1 were older compared to patients in group 2 but the difference was not statistically significant (P = 0.052). All patients presented with New York Heart Association (NYHA) class III or IV. About 28% of the patients had diabetes mellitus and about 45% of the patients suffered from arterial hypertension. Forty percent of had dyslipidemia while 19% of the patients were smokers.

### Baseline echocardiographic characteristics

From the total study population, half of the patients had mitral regurgitation grade 3+ and the other half had mitral regurgitation grade 4+. Systolic pressure in the pulmonary artery was 40.4 ± 13.7 mmHg in group 1 and 41.1 ± 8.8 mmHg in group 2. Left atrium diameter was comparable among patients in group 1 and group 2 (47.2 mm and 45.4 mm, respectively). Mean value of left ventricle ejection fraction was 59.3% among patients in group 1 and 59.1% among patients in group 2. There were no significant differences among patients in the two study groups regarding baseline echocardiographic characteristics as shown in Table 1.

**Table 1:** Clinical and echochardiographic characteristics.

Characteristics	Group 1 (n = 18)	Group 2 (n = 52)	P value
Age, years	58.9 ± 13.9	65.0 ± 10.2	NS
Women, %	22.2	32.3	NS
Diabetes mellitus, %	27.5	28.5	NS
Arterial hypertension, %	46.0	44.2	NS
Dyslipidemia, %	38.5	41.5	NS
Smokers, %	16.4	21.6	NS
Chronic obstructive pulmonary disease, %	11.1	15.4	NS
Prior myocardial infarction, %	-	3.8	NS
Arrhythmia, %	38.8	40.3	NS
Body mass index, kg/m^2^	24.2 ± 3.8	24.7 ± 3.8	NS
Left ventricle ejection fraction, %	59.3 ± 4.9	59.1 ± 7.1	NS
Left atrium diameter, mm	47.2 ± 7.0	45.4 ± 6.7	NS
Pulmonary artery systolic pressure, mmHg	40.4 ± 13.7	41.1 ± 8.8	NS

NS - nonsignificant.

### Postoperative outcome

Data on the postoperative outcome of study patients are shown in Table 2. Eleven patients in group 1 had residual mitral regurgitation 1+/2+ while 7 patients had no residual mitral regurgitation. Seventeen patients in group 2 had residual mitral regurgitation 1+/2+ and 35 patients had no residual mitral regurgitation. The proportion of patients with residual mitral regurgitation 1+/2+ was higher among patients in group 1 as compared to patients in group 2 (61.1% vs. 32.7%, respectively; P = 0.034). In the analysis of logistic regression, in which residual mitral regurgitation was entered as a response variable, prolapse of anterior leaflet was identified as an independent predictor or residual mitral regurgitation 1+/2+ (odds ratio, 4.0; 95% confidence interval, 1.14 to 14.04; P = 0.03).

**Table 2:** Results/inhospital complications.

Results/Complications	Group 1 (n = 18)	Group 2 (n = 52)	P value
Residual mitral regurgitation 1+/2+, %	61.1	32.7	0.034
Reintervention, %	11.1	9.6	NS
Death, %	0	0	-
Need for inotropic drugs, n	7	21	NS
Intubation time, hours	12.6 ± 7.5	9.3 ± 7.0	NS
Length in intensive care unit, days	1.5 ± 0.8	1.1 ± 0.7	NS
Total hospital stay, days	9.2 ± 3.0	8.4 ± 3.2	NS

NS - nonsignificant.

No deaths occurred among patients included in this study. There were no differences among patients included in group 1 and patients included in group 2 regarding the need of reintervention, length of stay in intensive care unit and total hospital length of stay. None of the patients in group 1 and one patient in group 2 underwent re-operation for mitral valve replacement.

## Discussion

The superiority of valve sparing techniques as compared to valve replacement for the treatment of mitral regurgitation has been widely demonstrated with regard to more favourable mortality and morbidity rates [8]. Reconstructive procedures ensure a more effective and durable competency of the repaired mitral valve [8]. However, early experiences with mitral valve repair in the presence of anterior leaflet prolapse have been less favourable due to more complex mechanisms of valvular incompetence and more demanding repairing techniques. Prolapse of the anterior leaflet is more difficult to repair because it is hardly possible to perform a resection of the anterior leaflet. The height of the chordae underlying the anterior leaflet must be assessed and chords may be grossly elongated or ruptured and make the AML flail. Correction of anterior leaflet prolapse, can be more difficult than posterior leaflet repair; redundancy of the posterior leaflet lends itself to plication or segmental excision, but prolapsing portions of the anterior leaflet are often not redundant nor amenable to excision. Poor results after anterior leaflet resections have led to a variety of techniques including chordal transposition, chordal shortening, and chordal replacement. The aetiology and responsible mechanisms might be negative prognostic factors for the success of mitral valve repair and could lead to a higher incidence of recurrent mitral regurgitation and need for reintervention.

We studied patients who underwent repair of mitral valve for mitral regurgitation due to mitral valve prolapse. We found that patients with prolapse of the anterior leaflet had a higher proportion of residual mitral regurgitation 1+/2+ as compared to patients with prolapse of posterior leaflet after mitral valve repair. In hospital mortality in our group of patients was 0%. Duebener et al. studied 160 patients with mitral regurgitation due to mitral valve prolapse who underwent mitral valve repair and showed that there were no differences regarding mortality and need for reintervention among patients with anterior leaflet prolapse versus patients with posterior leaflet prolapse. Mortality was 4.2% and 0%, respectively in the group of patients with anterior and posterior leaflet prolapse in the above-mentioned study [9]. De Bonis et al. have demonstrated comparable long-term results after repair with different techniques of anterior leaflet prolapse versus posterior leaflet prolapse [10]. While Fukui et al. demonstrated in a population of 26 patients who were followed-up for 3.7 years a significant increase in the deterioration of residual mitral regurgitation after repair of anterior mitral leaflet as compared to the posterior mitral leaflet [11]. The reasons for the differences between the results of our study and the results of the abovementioned studies might be found in the different characteristics of patients included in theses studies, surgical techniques used, and duration of follow-up. In a recent analysis, we found that the presence of less than moderate residual mitral regurgitation after valve repair was associated with a non significant trend toward an increased incidence of early adverse events as compared to the absence of residual mitral regurgitation [12].

## Conclusion

Among patients undergoing surgical correction of mitral regurgitation due to mitral valve prolapse, repair of anterior leaflet was associated with a higher proportion of residual mitral regurgitation 1+/2+ as compared to the repair of posterior leaflet. The clinical significance of residual mitral regurgitation 1+/2+ in this patient population is not known.

## Competing interests

The authors declare that they have no competing interests.

## Authors' contributions

LS, AR, and SB conceived and designed the study; LS, AR, GT, CP, MR, and SM participated in patient selection and performed echocardiographic analysis; MG carried out the mitral valve repair; LS and AR gathered the data; LS, AR, AD, and SB performed the statistical analysis and interpreted the data; LS drafted the manuscript; AR, MG, GT, CP, MR, AD, SM, and SB critically revised the manuscript; AR and SB provided administrative, technical, or material support; SB supervised the study. All authors read and approved the final version of the manuscript.
